# Changes in the Soil Fungal Community in a Temperate Deciduous Forest at Different Altitudes in the Taihang Mountains

**DOI:** 10.3390/jof11110800

**Published:** 2025-11-10

**Authors:** Liu Yang, Jinhua Sun, Ting Wang, Shu Zhao, Qingxin Li, Xitian Yang, Lianfeng Shen, Guohang Tian, Feiyan Ren

**Affiliations:** 1 Forestry College, Henan Agricultural University, Zhengzhou 450046, China; 2 Postdoctoral Research Station of Landscape Architecture, Henan Agricultural University, Zhengzhou 450046, China; 3 College of Landscape Architecture & Art, Henan Agricultural University, Zhengzhou 450046, China

**Keywords:** soil fungal community, forest, soil chemical properties, different altitudes

## Abstract

Soil fungi play vital roles in the forest soil ecosystems through the nutrient cycle and organic substance decomposition, so the distribution of fungi at different altitudes has attracted increasing attention. However, their abundance, diversity, and community structure at different altitudes in temperate deciduous forests have rarely been studied. In this study, the fungal communities around two dominant trees (*Quercus aliena* var. *acutiserrata* and *Carpinus turczaninowii* Hance) in temperate deciduous forests at different altitudes (low altitude, medium altitude, high altitude) in the Taihang Mountains were identified via Illumina high-throughput sequencing according to the UNITE database. The soil chemical properties (soil pH value, soil available potassium, alkaline hydrolyzed nitrogen, soil available phosphorus contents, soil total nitrogen, and carbon contents) were also measured. The results revealed that the dominant genera around the tree species were *Russula*, *Tylopilus*, *Sebacina*, *Saitozyma*, *Mortierella*, *Amanita*, and *Descolea*. The highest relative abundance of fungi occurred at the lowest altitude. The species richness index and diversity index of fungi around *Carpinus turczaninowii* at low altitudes were the highest. The soil pH, available potassium content, and alkaline hydrolyzed nitrogen content played a crucial role in the composition and diversity of the fungal communities at different altitudes. Therefore, soil physicochemical properties were the important factors in forming fungi composition and diversity at different altitudes in the temperate forest.

## 1. Introduction

Forest ecosystems have functions such as conserving water sources, maintaining soil and water, and regulating climate, providing abundant production and living resources for humans [1]. Fungi are important decomposers in forest soil and degrade large amounts of trees aboveground and root litter every year. The core role of fungi in forest ecosystems is to decompose organic matter, converting dead plant and animal residues (such as dead wood and fallen leaves) into inorganic substances (such as carbon dioxide, water, and inorganic salts), promoting nutrient cycling and material regeneration. In the soil, fungi are classified into pathogenic decomposers and symbionts based on their functions, and have been reported to regulate nutrient cycling [2,3]. The composition and diversity of fungal communities are influenced by both biotic and abiotic factors, such as host plant species [4,5], soil physicochemical and biological properties [6,7,8,9,10], and geographical factors [11]. Altitude is an important environmental factor that impacts biological diversity in mountain areas. There are significant differences in the site conditions of plants growing at different altitudes, such as sunlight, moisture, temperature, and soil properties [11].

The Taihang Mountains have important ecological functions, which effectively block dust, conserve water, and protect biodiversity [12]. Forest ecosystems have been degraded due to the long-term impact of human deforestation; therefore, it is an important ecological restoration area [13]. Microbial technology is widely applied as an important vegetation restoration measure [14]. However, the composition and diversity of fungal communities in the area are still unclear [15,16]. Different altitude regions provide different conditions, such as light, temperature, and moisture. Therefore, altitude likely substantially impacts fungal communities.

*Carpinus turczaninowii* Hance and *Quercus aliena* var. *acutiserrata*, which belong to the *Betulaceae* and *Fagaceae* families, respectively, are essential components of boreal temperate forests in the Taihang Mountains [17]. Through numerical advantage, ecological niche dominance, and functional irreplaceability, they play a key controlling role in the formation of community structure and environment [18]. As widespread species, *Carpinus turczaninowii* Hance and *Quercus aliena* var. *acutiserrata* also have strong sprouting ability; are resistant to drought and barrenness; have excellent soil protection to avoid erosion, slope protection, soil and water conservation, and fire prevention functions; they are also important high-quality bioenergy tree species [18]. However, the soil fungal community structure of the dominant tree species at different altitudes is still unclear.

While the driving factors of fungal communities have been extensively studied in various systems, the important factors influencing the community structure and diversity of fungi in the forest ecosystems of the Taihang Mountains have not yet been explored. These studies have provided a better understanding of the effect of altitudes on the soil fungal community through tree species and soil properties. In this work, we propose the following hypotheses: (1) The fungal community and structure of two tree species vary with altitude. (2) The richness of fungal communities is closely related to soil properties.

## 2. Materials and Methods

### 2.1. Site Description

**Figure 1:**
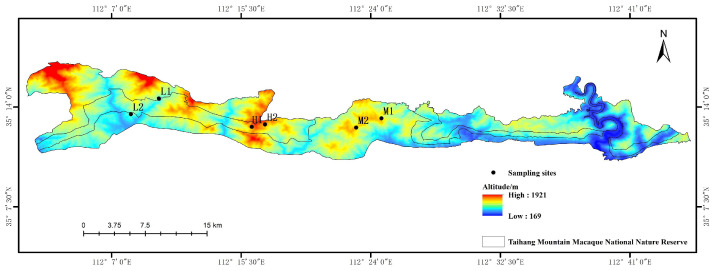
Study area and sampling sites at different altitudes. L1 and L2: low altitude; M1 and M2: medium altitude; H1 and H2: high altitude.

The study was conducted in the Taihang Mountains (34°54′–35°16′ N, 112°02′–112°52′ E) in the northwestern part of Henan Province, China. The layout of sampling points is shown in Figure 1. Three altitude sites, 600~900 m (low altitude), 901~1200 m (medium altitude), and 1201~1500 m (high altitude), were selected in the mountains. Six independent replications (25 m × 25 m) (two tree species with three replications for each sampling plot) were conducted at each altitude (Figure 1). The tree species included *Carpinus turczaninowii* Hance and *Quercus aliena* var. *acutiserrata*.

There are mountains in the northwest, hills in the southeast, and plains in the middle and east of the Taihang Mountains. The region has a warm temperate continental monsoon climate, with a mean annual temperature of 14.5 °C and a mean annual precipitation of 567.9 mm. The research area belongs to a mixed forest of deciduous broad-leaved forest and coniferous forest. In the west, except for a small part of the original forest near Aobei Mountain, the remaining forests are mostly miscellaneous wood forests of secondary oak forests. The limestone area is in the East, where there are small amounts of pine and cypress trees, and the remaining areas are mostly miscellaneous wood forests and shrubs, with a forest coverage rate of 48%.

### 2.2. Soil Sample Collection

We collected 18 soil samples (3 altitude levels, 2 tree species, and 3 replications). The small amount of soil (0~20 cm) in close contact with the roots of the two tree species was collected at each sampling plot on 14 July 2023. The temperature was 28 °C, the soil temperature was 30 °C, and the soil moisture content was 15%. After soil impurities such as stones, glass, and tree branches were removed, a 500 g soil sample was preserved. The soil samples were stored at 4 °C and divided into two parts: one for analyzing the composition and diversity of soil fungal communities through high-throughput sequencing methods, and the other for analyzing the soil’s physical and chemical properties.

### 2.3. Soil Fungal Community Composition and Diversity

MP Biomedicals Fast DNA (Santa Ana, CA, USA) was used to extract DNA from soil. A total of 0.3–0.5 g of soil was weighed, glass beads and suspension buffer (Buffer C1) and vortex were added and shaken for 3–5 min to break the soil structure. Lysis buffer C3 was added and glass beads were rubbed, and then DNA was released in a 70 °C water bath for 10 min. Subsequently, the DNA was precipitated with isopropanol, and impurities were removed by centrifugation. The decay buffer (Buffer R2) and adsorbent (magnetic beads) were used to adsorb humic acid to avoid interference in subsequent experiments. The supernatant was transferred to a silica gel purification column, the ethanol and centrifuge were washed to remove residual impurities, and finally, a DNA solution was obtained. High-quality and intact genomic DNA is a prerequisite for library amplification and construction. The integrity of the genomic DNA was detected with agarose gel electrophoresis. The electrophoretic bands were clearly visible without obvious degradation. The quality of the genomic DNA was examined by A Nanodrop 2000 (Thermo Fisher Scientific, Wilmington, NC, USA); the concentration and total amount were more than 20 ng/µL and 500 ng, respectively, and the OD260/280 ratio ranged from 1.8 to 2.0.

High-fidelity PCR amplification of the qualified sample detection areas with 3 replicate experiments was performed, and a positive control is the standard fungal genomic DNA mixture. The amplification primers were determined based on the selected detection area, and the primer pairs (forward, F/reverse, R) were “F = CTTGGTCATTTAGAGGAAGTAA, R = GCTGCGTTCTTCATCGATGC”. The target region was ITS1, and the PCR cycling conditions included 94 °C, 2 min; (94 °C, 30 s, 55 °C, 30 s, 72 °C, 1 min) * 25 or 30 cycles; 72 °C, 10 min; 4 °C, hold. Agarose gel electrophoresis was used to detect whether the amplification product is single and specific. The three parallel amplification products from the same sample and equal volumes of Agencourt AMPure XP nucleic acid purification magnetic beads were added to each sample in order to purify the products. Primers were used with index sequences, and specific tag sequences were introduced to the end of the library through high-fidelity PCR. This enabled downstream sequencing to mix multiple samples, and the samples were distinguished with different tag sequences through subsequent bioinformatics processing. The agarose gel electrophoresis was used to detect the amplified product, and the nucleic acid purification magnetic beads were used to obtain an original library of the samples.

According to the preliminary quantitative results of agarose gel electrophoresis, the concentration of the sample library with its respective index tags was diluted appropriately, and then the library was quantified accurately using Qubit (Thermo Fisher Scientific, Singapore). According to the sequencing flux requirements of different samples, the samples were mixed according to the corresponding molar proportions. The mixed library was analyzed by an Agilent 2100 Bioanalyzer (Agilent component, Santa Clara, CA, USA) to determine the size of the inserted fragments in the sequencing library. The insert size was 200–300 bp, and the library concentration was accurately quantified. The library was sequenced using the MiSeq platform and a 2 × 250 bp double-ended sequencing strategy, followed by bioinformatics analysis.

### 2.4. Soil Properties

A pH meter was used to measure the soil pH values at a 1:2.5 soil/water ratio (*w*/*v*). The soil total nitrogen and carbon concentrations were measured by an elemental analyzer (Elementar Americas, Inc., Mt. Laurel, NJ, USA). The soil organic matter content was analyzed by the external heating potassium dichromate volumetric method. The soil available phosphorus content was measured by the molybdenum-antimony colorimetric method, and the soil alkaline hydrolyzed nitrogen content was determined by the external diffusion method [19].

### 2.5. Statistical Analysis

RDP (Ribosomal Database Project) was used for fungal classification and identification through 28S rRNA gene analysis. The alpha diversity indices, such as Shannon and Simpson indices, were calculated by the software Bio-Dap (http://hi.baidu.com/kuanjin8309/blog/item/547cd4cb5b30db43f31fe7f5.html, 1 June 2025). The Chao1 and ACE indices were used to determine fungal richness. A permutational multivariate analysis of variance (PERMANOVA, Adonis function) using the Bray–Curtis distance, accompanied by a test for homogeneity of multivariate dispersion (betadisper), was conducted. The effects of altitude and tree species on fungal community characteristics and soil properties were analyzed through two-way ANOVA. The LSD test was used to test for significant differences among different altitudes at *p* < 0.05.

Pearson correlation analysis with a two-tailed test (significance level *p* < 0.05) between the soil properties and the relative abundances of the dominant fungal communities was performed by SPSS 25.0. Redundancy analysis (RDA) was used to analyze the relationship between fungal community composition and environmental factors by R Language 4.3.2, and the significance of the effect is tested by the K-square test.

## 3. Results

### 3.1. Soil Properties Change 

The soil pH values of the two tree species increased with increasing altitude (Table 1), and the soil pH values at medium altitudes were significantly lower than those at high altitudes. The rhizosphere soil available potassium content, alkaline hydrolyzed nitrogen content, available phosphorus content, total nitrogen content, and total carbon content at low altitudes were highest. The soil alkaline hydrolyzed nitrogen content around *Quercus aliena* var. *acutiserrata* was significantly greater than that around *Carpinus turczaninowii* Hance at low and medium altitudes. Regardless of altitude, the soil available phosphorus, total nitrogen, and total carbon contents around *Quercus aliena* var. *acutiserrata* were significantly greater than those around *Carpinus turczaninowii* Hance.

### 3.2. Community Composition and Relative Abundance of Fungi

**Figure 2:**
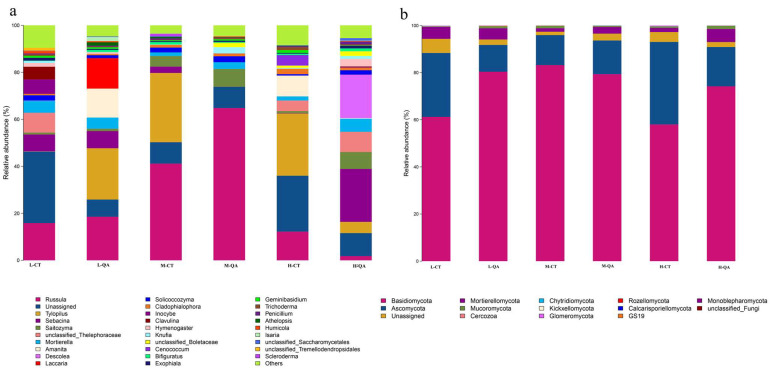
Fungal community composition at the generic (**a**) and phylum (**b**) levels. L-CT, *Carpinus turczaninowii* Hance at low altitude; L-QA, *Quercus aliena* var. *acutiserrata* at low altitude; M-CT, *Carpinus turczaninowii* Hance at medium altitude; M-QA, *Quercus aliena* var. *acutiserrata* at medium altitude; H-CT, *Carpinus turczaninowii* Hance at high altitude; H-QA, *Quercus aliena* var. *acutiserrata* at high altitude. The same annotations are used in the following figures.

**Figure 3:**
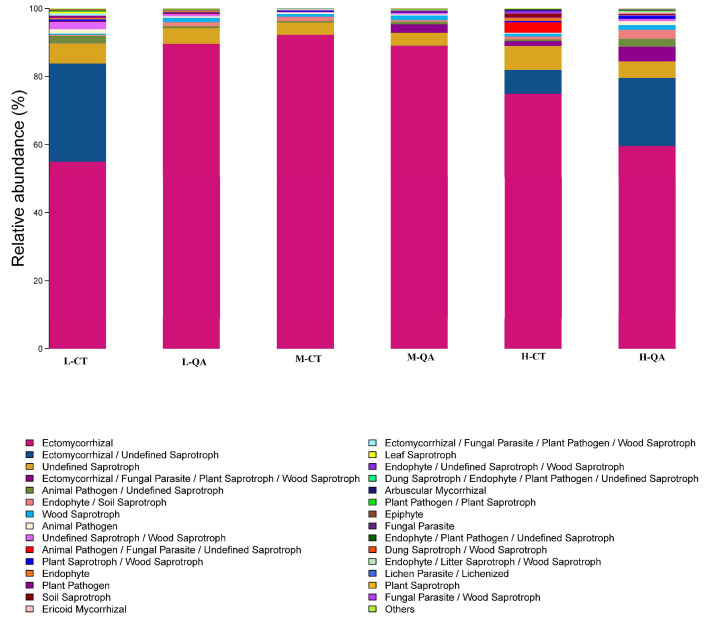
The relative abundance of fungal groups.

At the genus level, *Russula*, *Tylopilus*, *Sebacina*, *Saitozyma*, *Mortierella*, *Amanita*, and *Descolea* were the dominant fungi in the research region (total relative abundances were more than 70%). However, *Amanita* and *Sebacina* were dominant around *Quercus aliena* var. *acutiserrata* at high altitudes (Figure 2a, data shown in Table S1). *Basidiomycota* and *Ascomycota* were the main phyla at medium altitudes (more than 70%) (Figure 2b, data shown in Table S2). At low or high altitudes, *Ascomycota* were more likely to bind with *Carpinus turczaninowii* Hance. Ectomycorrhizal fungi represented most of the observed fungal groups (more than 50%) in both tree species at high altitudes, and represented more than 80% at medium altitudes (Figure 3, data shown in Table S3).

### 3.3. Richness and Diversity of Fungi Among Different Altitudes and Tree Species

**Figure 4:**
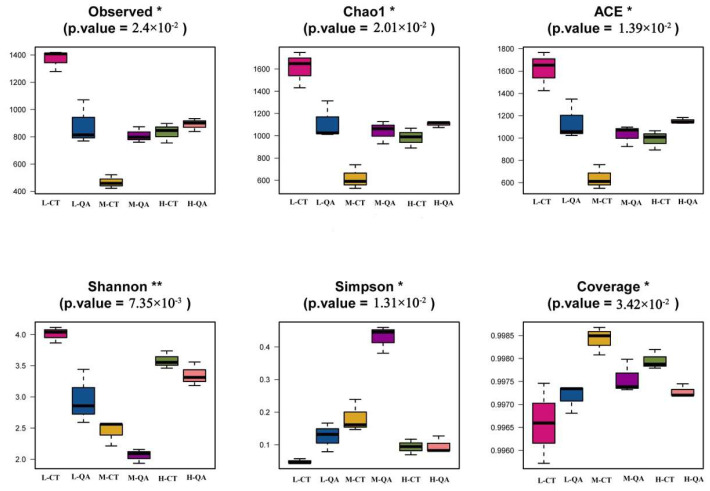
Alpha diversity of soil fungi in two oak species. * indicates that the variables are significantly correlated at the 0.05 level. ** indicates that the variables are significantly correlated at the 0.001 level.

A total of 1,398,377 clean reads were obtained from the rhizosphere soils of the two tree species at three altitudes, after which 1,499,743 raw reads were obtained (ranging from 157,619 to 296,177). The fungal OTUs of *Carpinus turczaninowii* Hance at low altitudes were highest at different altitudes, and the fungal OTUs of *Carpinus turczaninowii* Hance at medium altitudes were lowest at the super genus levels (Table 2). The interaction between tree species and altitude affects the soil fungal community diversity indices. The soil fungal Chao1 index and the ACE richness index were highest for *Carpinus turczaninowii* Hance at low altitudes and lowest for *Carpinus turczaninowii* at intermediate altitudes (Figure 4). The Shannon diversity index of *Carpinus turczaninowii* was highest at low altitudes, but that of *Quercus aliena* var. *acutiserrata* was highest at high altitudes. The Simpson diversity indices of *Carpinus turczaninowii* and *Quercus aliena* var. *acutiserrata* were highest at medium altitudes.

**Figure 5:**
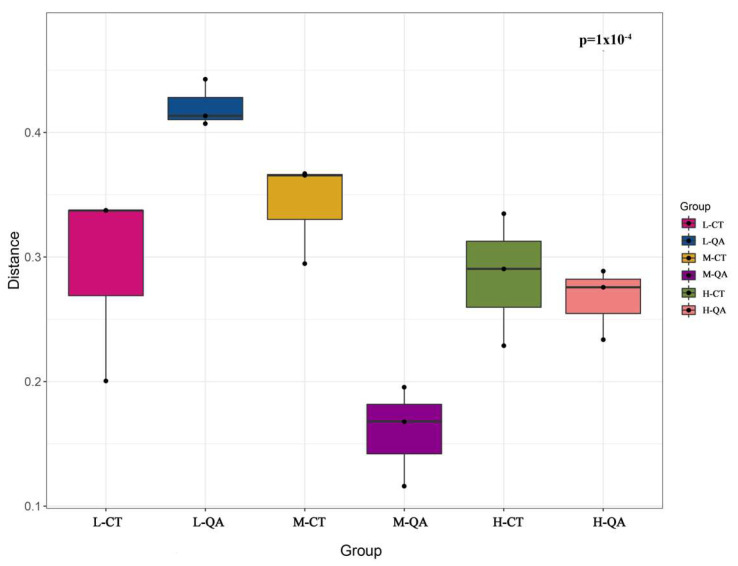
A permutational multivariate analysis of all samples at different altitudes.

The distance between different samples was significant (Figure 5), indicating that the differences in the fungal diversity of two tree species among the different altitudes were significant. The close proximity of soil fungal diversity among the same tree species indicates that tree species have a significant impact on fungal community composition.

### 3.4. Relationships Between the Soil Properties and Fungal Communities

As shown in Table 3, the relative abundance of *Russula* was significantly negatively correlated with the soil total nitrogen content (*p* < 0.05) and significantly positively correlated with the soil alkaline hydrolyzed nitrogen content. The relative abundance of *Tylopilus* was significantly negatively correlated with the available potassium content (*p* < 0.05). The relative abundance of *Sebacina* was significantly negatively correlated with the soil alkaline hydrolyzed nitrogen content (*p* < 0.001). The relative abundance of *Saitozyma* was significantly positively correlated with the soil alkaline hydrolyzed nitrogen content (*p* < 0.001). The relative abundance of *Mortierella* was significantly positively correlated with the soil pH value and total nitrogen content (*p* < 0.05) and significantly negatively correlated with the soil alkaline hydrolyzed nitrogen content (*p* < 0.001). Therefore, the soil chemical properties had a significant effect on the fungal community, and the soil pH value, available potassium content, and alkaline hydrolyzed nitrogen content were the main factors influencing the fungal community structure.

**Figure 6:**
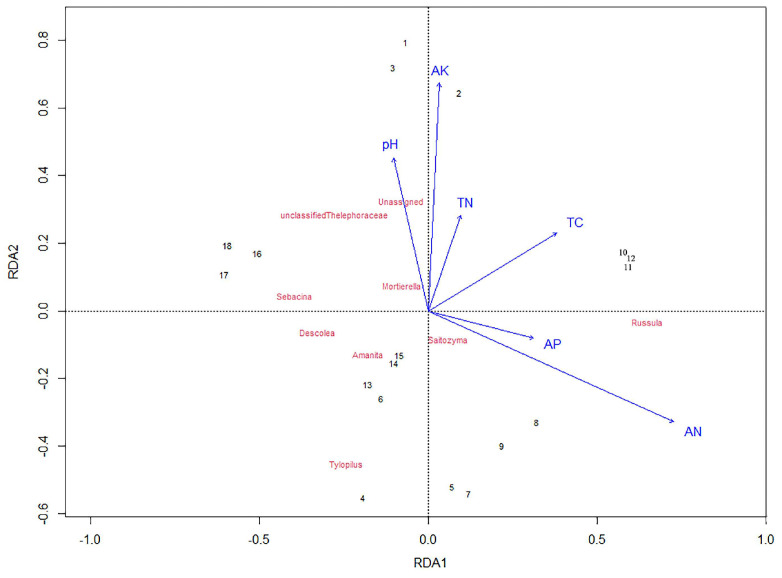
Redundancy analysis (RDA) for soil fungal diversity and soil properties. AK, available potassium content; AP, available phosphorus content; AN, alkaline hydrolyzed nitrogen content; TN, total nitrogen content; TC, total carbon content. 1~3 indicate *Carpinus turczaninowii Hance* at low altitude; 4~6 indicate *Quercus aliena* var. *acutiserrata* at low altitude; 7~9 indicate *Carpinus turczaninowii* Hance at medium altitude; 10~12 indicate *Quercus aliena* var. *acutiserrata* at medium altitude; 13~15 indicate *Carpinus turczaninowii* Hance at high altitude; 16~18 indicate *Quercus aliena* var. *acutiserrata* at high altitude.

RDA shows that the soil pH, available potassium content, alkaline hydrolyzed nitrogen content, available phosphorus content, total nitrogen content, and total carbon content play important roles in determining the fungal community structure (Figure 6). *Russula* and *Saitozyma* were significantly impacted by the soil available phosphorus content and alkaline hydrolyzed nitrogen content. *Unassigned*, *Sebacina*, *unclassified_Thelephoraceae*, and *Mortierella* were significantly correlated with the soil pH. The variance results from the principal component analysis revealed that the contribution rates of the first and the second components were 16.78% and 8.28%, respectively (*p* < 0.05, Table 4). The fungal communities were divided into four groups (Table 5), which indicated that altitude and tree species affected the soil fungal community structure.

## 4. Discussion

### 4.1. Response of Soil Properties to Different Altitudes

Soil is an important site for material cycling in forest ecosystems, and soil quality is influenced by altitude, climatic characteristics, aboveground plants, and microorganisms [20,21]. In our study, pH decreased with increasing altitude. The soil in the research area was acidic, ranging from 4.53 to 5.36 (Table 1). Soil acidity mainly comes from the acidic secretions of tree roots and organic acids secreted by fungi [22]. The variation in soil pH with altitude has been confirmed by previous studies [23].

The available potassium concentration, available nitrogen concentration, available phosphorus concentration, total nitrogen content, and carbon content of the rhizosphere soil at low altitudes were significantly greater than those of the rhizosphere soil at medium altitudes or high altitudes (Table 1). The altitude affects the distributions of water, heat, and light in mountainous forests, directly or indirectly affecting soil properties [24]. Precipitation and temperature, which are regulated by altitude, play important roles in the accumulation of plant biomass, leading to the feedback of fallen leaves and root exudates into the soil and affecting the nutrient and organic matter contents of the soil [25]. In a previous study, altitude was negatively related to organic matter and nitrogen [26]. Moreover, Soil organic matter often limits the soil nutrient support, leading to reduced yields in mountainous areas [26]. The low temperatures at relatively high altitudes resulted in less active microbes in the topsoil, which in turn reduced the microbial decomposition of soil organic matter and minerals [27]. Erosion at high altitudes results in the loss of many soil nutrients and organic matter [28].

In this study, the available phosphorus, alkali hydrolyzable nitrogen concentration, total nitrogen content, and total carbon content in the rhizosphere soil of *Quercus aliena* var. *acutiserrata* were significantly greater than those in the rhizosphere soil of *Carpinus turczaninowii* Hance (Table 1). This may be due to the different fungal communities’ composition around the roots of different tree species, and their varying abilities to degrade organic matter and transform nutrients.

### 4.2. Varieties in the Soil Fungal Community Composition at Different Altitudes

In this study, the soil fungi community composition (e.g., ectomycorrhizal fungi and saprophytic fungi) decreased with increasing altitude. *Basidiomycota* and *Ascomycota* were the two dominant phyla of the soil fungal communities in the region (Figure 2b), which was consistent with previous research findings [11,29]. In our study, *Russula*, *Tylopilus*, *Sebacina*, *Saitozyma*, *Mortierella*, *Amanita*, and *Descolea*. *Tuber*, *Russula*, and *Sordariales* are the dominant ectomycorrhizal fungi in *Fagaceae* tree species [30]. Both *Russula* and *Amanita* are late successional species in a previous study [31,32]. *Inocybe*, *Russula*, and *Tomentella* were found to be dominant at oak forests [33]. *Fagaceae* plants are associated with ECM fungi and can form a large mycelial network to participate in the nutrient cycle and energy metabolism of the host [30].

A decrease in temperature with increasing altitude is a common environmental gradient. Which controlled the physiological activities of organisms such as plants, animals, and microorganisms, their distribution in high-altitude areas is also restricted [34,35]. The relative abundances of *Basidiomycota* and *Ascomycota* exhibited different altitudinal patterns (Figure 2b), which indicates that there was niche differentiation among the soil fungal taxa along an altitudinal gradient [36]. Soil fungal communities dominated by ectomycorrhizal fungi exhibited a hump-shaped pattern with an altitudinal gradient, which was consistent with the results of the present study.

### 4.3. Response of the Soil Fungal Community to Soil Properties at Different Altitudes

Soil properties are important environmental factors that affect fungal activity. In this study, the soil pH, available potassium, and alkaline hydrolyzed nitrogen were the main factors influencing fungal community structure (Figure 6). This finding is consistent with previous research findings [37,38]. Soil pH is the most dominant driver of fungal richness, phylodiversity, and evenness [39]. The diversity of a fungal community decreases with increasing pH [40]. However, a lower pH acts as a pressure for most fungal communities, which is beneficial for maintaining the community but not conducive to its prosperity [41]. Compared with prokaryotes and bacteria, fungi are less affected by soil pH [42,43]. Soil pH can alter N, P, and K availability or impose physiological limitations on affected fungal community structure [44]. In the previous study, soil pH, soil organic matter, and alkaline hydrolyzed nitrogen are the main driving factors for forming ectomycorrhizal fungal community composition and diversity along the urban–rural gradient [30]. Soilalkaline hydrolyzed nitrogen could effectively prevent soil nitrogen loss and maintain soil fertility [45]. Most ectomycorrhizal fungi were found to prefer NH_4_^+^-N during growth [46]. In addition, different soil physicochemical properties affected the survival and growth of fungi [47].

## 5. Conclusions

In conclusion, an analysis of the fungal communities revealed that the fungal richness and diversity of the rhizosphere soil in temperate forests were influenced by the interaction between tree species and altitude, and the soil nutrient content decreased with increasing altitude. The main soil fungi in the research region were *Russula, Tylopilus*, *Sebacina*, *Saitozyma*, *Mortierella*, *Amanita*, and *Descolea*. The soil pH value, available potassium content, and alkaline hydrolyzed nitrogen content were the most important drivers among edaphic factors that determine the soil fungal community composition. These findings strengthened our knowledge of belowground biodiversity at different altitudes in temperate forests.

## Figures and Tables

**Table 1:** Soil chemical properties of the two dominant trees at different altitudes.

Treatments	pH	AK (mg/kg)	AN (mg/kg)	AP (mg/kg)	TN (%)	TC (%)
L-CT	4.79 ± 0.03 c	114.04 ± 1.83 a	133.28 ± 1.20 b	2.36 ± 0.05 c	0.096 ± 0.00 a	0.85 ± 0.01 a
L-AQ	4.77 ± 0.15 c	76.04 ± 0.08 bc	161.66 ± 1.22 a	5.32 ± 0.29 a	0.096 ± 0.00 a	0.86 ± 0.06 a
M-CT	4.53 ± 0.09 d	38.27 ± 0.28 e	87.41 ± 1.02 e	1.36 ± 0.1 d	0.073 ± 0.01 b	0.773 ± 0.06 b
M-AQ	4.88 ± 0.05 c	77.18 ± 1.12 b	115.77 ± 0.50 c	4.16 ± 0.03 b	0.084 ± 0.00 c	0.87 ± 0.01 a
H-CT	5.36 ± 0.07 a	75.09 ± 0.24 c	103.17 ± 0.53 d	0.18 ± 0.18 e	0.064 ± 0.01 c	0.66 ± 0.06 c
H-AQ	5.12 ± 0.10 b	57.42 ± 0.59 d	81.34 ± 0.09 f	1.25 ± 0.24 d	0.075 ± 0.00 d	0.75 ± 0.02 b

Note: L-CT, *Carpinus turczaninowii* Hance at low altitude; L-QA, *Quercus aliena* var. *acutiserrata* at low altitude; M-CT, *Carpinus turczaninowii* Hance at medium altitude; M-QA, *Quercus aliena* var. *acutiserrata* at medium altitude; H-CT, *Carpinus turczaninowii* Hance at high altitude; H-QA, *Quercus aliena* var. *acutiserrata* at high altitude; AK, available potassium content; AP, available phosphorus content; AN, alkaline hydrolyzed nitrogen content; TN, total nitrogen content; TC, total carbon content. Different lowercase letters represent significant differences between different treatments.

**Table 2:** Soil fungal OTUs at different levels of the two trees at the three altitudes.

	Superkingdom	Phylum	Class	Order	Family	Genus	Species
L-CT	1364 ± 44.54 a	795 ± 41.53 a	620 ± 34.84 a	594 ± 34.07 a	538 ± 30.81a	483 ± 27.06 a	377 ± 16.15 a
L-QA	882 ± 93.08 b	548 ± 55.17 b	451 ± 49.96 b	437 ± 46.61 b	397 ± 44.04 b	357 ± 39.34 b	286 ± 54.07 b
M-CT	466 ± 28.26 c	342 ± 19.68 c	291 ± 18.22 c	277 ± 16.20 c	254 ± 15.30 c	236 ± 13.87 c	196 ± 11.72 c
M-QA	809 ± 33.58 b	565 ± 21.37 b	464 ± 14.75 b	441 ± 14.62 b	397 ± 10.97 b	359 ± 9.54 b	295 ± 6.03 b
H-CT	832 ± 41.53 b	516 ± 34.95 b	415 ± 29.98 b	395 ± 29.30 b	350 ± 24.18 b	321 ± 22.84 b	260 ± 20.43 b
H-QA	886 ± 26.59 b	561 ± 17.89 b	459 ± 16.01 b	441 ± 13.50 b	403 ± 8.95 b	364 ± 9.35 b	284 ± 8.41 b

Note: L-CT, *Carpinus turczaninowii* Hance at low altitude; L-QA, *Quercus aliena* var. *acutiserrata* at low altitude; M-CT, *Carpinus turczaninowii* Hance at medium altitude; M-QA, *Quercus aliena* var. *acutiserrata* at medium altitude; H-CT, *Carpinus turczaninowii* Hance at high altitude; H-QA, *Quercus aliena* var. *acutiserrata* at high altitude. Different lowercase letters represent significant differences between different treatments.

**Table 3:** Correlation coefficients between dominant fungal species at the genetic level and soil properties.

	*Russula*	*Unassigned*	*Tylopilus*	*Sebacina*	*Saitozyma*	*Unclassified_* *Thelephoraceae*	*Mortierella*	*Amanita*	*Descolea*
pH	−0.2 (0.427)	0.251 (0.316)	−0.436 (0.07)	0.307 (0.215)	−0.328 (0.183)	0.256 (0.305)	0.635 (**)	−0.045 (0.861)	−0.037 (0.885)
AK	−0.155 (0.540)	0.718 (**)	−0.514 (*)	−0.117 (0.643)	−0.474 (*)	0.388 (0.111)	0.383 (0.117)	0.078 (0.759)	−0.292 (0.240)
AN	0.92 (**)	−0.502 (*)	0.078 (0.758)	−0.641 (**)	0.75 (**)	−0.793 (**)	−0.497 (**)	−0.091 (0.721)	−0.511 (*)
AP	0.378 (0.122)	−0.425 (0.079)	−0.251 (0.315)	−0.102 (0.686)	0.017 (0.946)	−0.460 (0.055)	0.294 (0.237)	0.286 (0.250)	−0.295 (0.235)
TN	0.037 (0.885)	0.019 (0.942)	−0.349 (0.156)	0.069 (0.786)	−0.234 (0.349)	−0.001 (0.996)	0.494 (*)	0.058 (0.821)	−0.216 (0.389)
TC	−0.487 (*)	−0.927 (**)	−0.854 (**)	−0.795 (**)	−0.851 (**)	−0.761 (**)	0.923 (**)	−0.678 (*)	−0.91 (**)

Note: The data outside the brackets indicate the correlation coefficient, whereas the data inside the brackets indicate the significance level of the correlation. * indicates that the variables are significantly correlated at the 0.05 level. ** indicates that the variables are significantly correlated at the 0.001 level. AK, available potassium content; AP, available phosphorus content; AN, alkaline hydrolyzed nitrogen content; TN, total nitrogen content; TC, total carbon content.

**Table 4:** Variance for principal component analysis.

	Variance	F	*p*
RDA1	0.1137	16.78	0.001 ***
RDA2	0.0561	8.28	0.013 *
RDA3	0.0254	3.75	0.304
RDA4	0.0161	2.38	0.411
RDA5	0.0063	0.92	0.635
RDA6	0.0002	0.04	0.996

Note: RDA1~RDA6 indicate the main environmental factors influencing the fungal community. “Variance” indicates the differences between principal components; F indicates the chi-square test value; *p* indicates the significance level; *** indicates *p* < 0.001; * indicates *p* < 0.05.

**Table 5:** Significant variance analysis of the eigenvalues.

	Variance	F	*p*
pH	0.029	4.32	0.007 **
AK	0.037	5.42	0.003 **
AN	0.103	15.25	0.001 ***
AP	0.026	3.88	0.013 *
TN	0.002	0.31	0.907
TC	0.02	2.95	0.036 *

Note: AK, available potassium content; AP, available phosphorus content; AN, alkaline hydrolyzed nitrogen content; TN, total nitrogen content; TC, total carbon content. *** indicates that the variables impact significantly at the 0.001 level; ** indicates that the variables impact significantly at the 0.01 level; * indicates that the variables impact significantly at the 0.05 level.

## Data Availability

The original contributions presented in this study are included in the article/Supplementary Material. Further inquiries can be directed to the corresponding authors.

## Supplementary Materials

The following supporting information can be downloaded at https://www.mdpi.com/article/10.3390/jof11110800/s1. Table S1: The fungi relative abundance at the phylum level. Table S2: The fungi relative abundance at the genus level. Table S3: The relative abundance of fungal groups.
