# Supplementary material for: Changes in the Soil Fungal Community in a Temperate Deciduous Forest at Different Altitudes in the Taihang Mountains
Source: J Fungi (Basel). 2025 Nov 10;11(11):800. doi: 10.3390/jof11110800 (PMC12653258; doi:10.3390/jof11110800)
Supplement: Supplementary file 1 [file jof-11-00800-s001.zip › jof-3928807-supplementary.pdf]

Table S1 The relative abundance of fungi at phylum level

Taxon	L-CT	L-QA	M-CT	M-QA	H-CT	H-QA	p
Glomeromycota	0.001	0.0001	0	0	0.003	1.42E-05	2.29E-07
Ascomycota	0.271	0.113	0.127	0.143	0.350	0.167	9.25E-06
Basidiomycota	0.612	0.804	0.832	0.794	0.580	0.742	1.93E-05
Mucoromycota	0.002	0.009	0.011	0.004	0.006	0.014	0.001
Mortierellomycota	0.051	0.048	0.015	0.029	0.018	0.056	0.005
Kickxellomycota	0.001	6.40E-05	1.42E-05	7.47E-05	2.85E-05	3.56E-06	0.006
Rozellomycota	0.0002	0.0004	0.0002	0.0003	0.0001	0.0001	0.006
Cercozoa	0.0013	0.002	0.0001	5.69E-05	2.13E-05	0.00015	0.007
Chytridiomycota	0.0009	0.0002	8.18E-05	0.0010	0.0006	0.00044	0.017
unclassified_Fungi	0	0	0	0	0	1.78E-05	0.033
Mortierellomycota	0.05	0.048	0.015	0.029	0.018	0.056	0.005

Note: L-CT, *Carpinus turczaninowii Hance* at low altitude; L-QA, *Quercus aliena var. acutiserrata* at low altitude; M-CT, *Carpinus turczaninowii Hance* at medium altitude; M-QA, *Quercus aliena var. acutiserrata* at medium altitude; H-CT, *Carpinus turczaninowii Hance* at high altitude; and H-QA, *Quercus aliena var. acutiserrata* at high altitude. p is the test value.(The same as Table S2 and Table S3)

Table S2 The relative abundance of main fungi at genus level

Taxon	L-CT	L-QA	M-CT	M-QA	H-CT	H-QA	p
Russula	0.158	0.186	0.411	0.648	0.122	0.018	2.75E-06
Tylopilus	0.001	0.218	0.295	0.0001	0.265	0.048	0.0003
Sebacina	0.071	0.073	0.027	0.0003	0.002	0.225	5.63E-06
Saitozyma	0.008	0.0099	0.045	0.076	0.008	0.072	2.84E-06
unclassified_Thelephoraceae	0.084	6.05E-05	2.85E-05	0.0002	0.045	0.086	0.0002
Mortierella	0.051	0.0476	0.015	0.029	0.018	0.056	0.005
Amanita	0.0008	0.123	7.83E-05	4.98E-05	0.088	0.003	5.00E-07
Descolea	6.40E-05	5.34E-05	1.42E-05	1.42E-05	7.11E-06	0.185	6.68E-07
Lactarius	9.25E-05	4.62E-05	5.34E-05	2.49E-05	0.006	0.0001	0.006
Cladophialophora	0.0059	0.0055	0.012	0.011	0.023	0.011	0.035
Inocybe	0.061	0.0001	0.0001	0.0003	3.56E-05	0.003	5.27E-05
Clavulina	0.054	0	3.56E-06	0.0002	0	0.002	4.51E-05
Hymenopellis	0.0002	2.13E-05	0	1.42E-05	3.56E-06	1.42E-05	0.0001
Cenococcum	0.0002	0.0001	0	0	0.045	0.0003	1.45E-07
Bifiguratus	0.002	[illegible]	[illegible]	0.004 / *0.00995*	[illegible]	[illegible]	0.002 / *0.01196*

Table S3 The relative abundance of fungal groups

Taxon	L-CT	L-QA	M-CT	M-QA	H-CT	H-QA	p
Ectomycorrhizal / Fungal Parasite /	0.006	0.0002	0.0003	0.0013	5.07E-06	0.0003	6.51E-08

Plant Pathogen / Wood Saprotroph							
Arbuscular Mycorrhizal	0.00043	0	0	0	0.00372	2.33E-05	1.12E-07
Endophyte / Litter Saprotroph / Wood Saprotroph	0	0	0	3.83E-05	0.00047	0.0014	8.90E-07
Animal Pathogen / Undefined Saprotroph	0.022	0.0065	0.0048	0.0077	0.0052	0.0236	2.28E-06
Fungal Parasite	0.00044	4.95E-05	9.26E-06	0.00012	0	0.00224	4.79E-05
Dung Saprotroph / Plant Saprotroph	0	0	2.31E-05	0	1.01E-05	0.00047	5.34E-05
Soil Saprotroph	0.0003	0.0009	0.0002	0.0003	0.009	0.0025	7.61E-05
Algal Parasite / Leaf Saprotroph / Wood Saprotroph	0.00047	3.09E-05	0	1.91E-05	5.25E-06	3.18E-05	7.72E-05
Ectomycorrhizal	0.549	0.892	0.922	0.89	0.749	0.597	8.63E-05
Leaf Saprotroph	0.0046	0.00058	0.00011	0.0011	0.00013	0.00121	8.78E-05
Endophyte / Soil Saprotroph	0.0029	0.012	0.013	0.005	0.009	0.026	0.00012
Ectomycorrhizal / Undefined Saprotroph	0.289	6.51E-05	5.49E-05	0.00044	0.07008	0.19883	0.00013
Undefined Saprotroph / Wood Saprotroph	0.0212	0.0073	0.00017	0.005	8.76E-05	0.006	0.0002
Animal Pathogen / Fungal Parasite / Undefined Saprotroph	0.0017	0.0022	0.0006	0.0007	0.0309	0.0005	0.0004
Ericoid Mycorrhizal	0.00067	0.00045	0.00312	0.0004	0.0006	0.0045	0.001
